# Postoperative alpha angle seems to be important for the achievement of clinical significance at a minimum 5-year follow-up after primary hip arthroscopy

**DOI:** 10.1093/jhps/hnad010

**Published:** 2023-05-09

**Authors:** Onur Gürsan, Onur Hapa, Dean K Matsuda, Selahaddin Aydemir, Mustafa Çeltik, Hakan Cici, Ahmet Emrah Acan

**Affiliations:** Department of Orthopedic Surgery, Dokuz Eylül University, Izmir 35340, Turkey; Department of Orthopedic Surgery, Dokuz Eylül University, Izmir 35340, Turkey; DISC Sports and Spine Center, Premier Hip Arthroscopy, Marina del Rey, CA 90292, USA; Department of Orthopedic Surgery, Dokuz Eylül University, Izmir 35340, Turkey; Department of Orthopedic Surgery, Dokuz Eylül University, Izmir 35340, Turkey; Department of Orthopedic Surgery, Democracy University, Izmir 35390, Turkey; Department of Orthopedic Surgery, Balıkesir University, Balıkesir 10145, Turkey

## Abstract

The purpose of the present study was to clarify whether there is an association of postoperative alpha value with functional scores or progression of osteoarthritis at X-rays at the midterm after arthroscopic treatment of femoroacetabular impingement (FAI) syndrome with femoral osteoplasty, labral repair or debridement and rim trimming. A retrospective review of prospectively gathered data from 2013 to 2017 was performed. All patients who underwent first-time unilateral hip arthroscopy for FAI resection with 5-year follow-up were included. Patient-reported outcomes included the modified Harris Hip Score (mHHS) and Visual Analog Scale for Pain (Pain VAS). The progression of osteoarthritis (Tönnis grade) and radiological parameters (alpha angle, lateral center-edge angle [LCEA] and head-neck offset) were evaluated. A receiver operating characteristic (ROC) analysis was used to evaluate the correlation between significant variables and achievement of patient-acceptable symptomatic state (PASS) and degree of osteoarthritis. We identified 52 patients with a minimum 5-year follow-up (average, 6.7 years). The average patient age was 33.9 ± 11.5 years. There were 19 (36.5%) female patients. The mHHS improved from 60.1 ± 13.4 before surgery to 86.8 ± 14 after surgery (*P* < 0.001). The Pain VAS decreased from 6.21 before surgery to 2 after surgery (*P* < 0.001). Overall, 69% achieved the PASS for mHHS. The ROC curve for postoperative alpha angle demonstrated acceptable discrimination between patients achieving a fifth-year PASS value and those who did not have an area under the curve of 0.72. Patients having a postoperative alpha angle of ≤48.3° achieved the fifth-year PASS value at a significantly higher rate than patients having a postoperative alpha angle of >48.3° (*P* = 0.002). The postoperative alpha angle is a predictor of the achievement of the fifth-year PASS value for the mHHS. A threshold of ≤48.3° had a sensitivity of 0.75 and a specificity of 0.69 to predict positivity.

Level of evidence IV

## INTRODUCTION

Hip arthroscopy provides successful treatment of the femoral-based deformity seen in cam impingement and acetabular-based abnormalities seen in pincer-type impingement [1]. For cam impingement, the alpha angle is the most common radiographic sign used for diagnosis [2–6]. A systematic review concluded that there was evidence that the correction of the alpha angle to <55° would result in improved outcomes [7]. Contrary to this, most studies report no association between postoperative alpha angle and patient-reported outcomes (PROs) [8–13]; additionally, the most recent consensus declared that more studies are needed to determine therapeutic thresholds that can be universally applied [14], while two recent studies reported evidence for the association of return to sports or PROs with postoperative alpha value [1, 15]. However, only the study by Monahan *et al*. [15] specifically reported a threshold value of 46° for the postoperative alpha angle which was associated with a higher return to sports.

The second controversial issue is whether this decrease in alpha angle leads to decreased development of hip osteoarthritis [16]. Most studies report no association between postoperative alpha angle and progression of osteoarthritis or theoretical benefits of correction of alpha angle or cam deformity on the prevention of osteoarthritis development [17, 18], while a recent study reported an increased arthroplasty conversion rate associated with higher postoperative alpha angle [19]. As a PRO model, the modified Harris Hip Score (mHHS) is a useful tool after hip surgery. Patient-acceptable symptomatic state (PASS) is one of the metrics that was developed to assess the clinical importance of PROs [20]. The purpose of this study is to clarify whether there is an association among postoperative alpha value, functional scores and progression of radiographic osteoarthritis. The primary outcome measure was to clarify whether there is a similar rate (65.8–72.1%) [6, 21, 22] of achievement of PASS value of hip score defined for the fifth year postoperatively. The secondary outcome measure is whether this value is related to the postoperative alpha value. The hypothesis is that there is a correlation between postoperative alpha value and functional hip score and change in Tönnis grade, especially when numerical data are categorized in a binary fashion like the achievement of a threshold value for PASS or not. There is a cutoff value for this angle below which patients would reach higher rates of PASS defined for the fifth year postoperatively and lower grades of hip osteoarthritis.

## MATERIALS AND METHODS

Fifty-two patients who had undergone hip arthroscopy for femoroacetabular impingement (FAI) between January 2013 and January 2017 were included in this study. The local ethics committee approval was obtained. Preoperative and postoperative subjective data and radiographic data were prospectively collected and retrospectively reviewed.

### Inclusion criteria

Patients were included if they underwent primary hip arthroscopy with a minimum follow-up of 60 months. FAI was diagnosed based on clinical symptoms and radiographic findings (alpha angle >55° for cam deformity using the Dunn 45° view [23, 24] and lateral center-edge angle [LCEA] ≥35°) [15, 25]. Surgery was indicated when there is persistent hip pain refractory to conservative treatment for at least 3 months. There were 89 patients who had a minimum 5-year follow-up. Patients were excluded if they had bilateral symptoms (*n* = 3), avascular necrosis (*n* = 5), advanced-level hip osteoarthritis (*n* = 4) (Tönnis Grade 3) [26], any previous ipsilateral hip surgery (*n* = 4), revision hip arthroscopy (*n* = 7) or incomplete radiographs (*n* = 1) or could not be reached (*n* = 13). Fifty-two patients were left for the analysis.

### Surgical procedure

The patient is placed supine on a hip arthroscopy–specific traction table to obtain appropriate hip distraction against a well-padded perineal post. A horizontal interportal capsulotomy is used to improve the visualization and access to the central compartment.

A 4.5-mm arthroscopic burr is used to perform acetabuloplasty. Degenerative labral tears or those with multiple cleavage planes were considered irreparable, and unstable flaps were selectively debrided. Tears that involved the base of the labrum with chondrolabral disruption were repaired using 1 to 3 suture anchors. Traction is then released, the peripheral compartment is entered and decompression of the cam deformity is performed and confirmed by intraoperative fluoroscopy and arthroscopic dynamic examination. The capsule was routinely left open at the end of the procedure.

### Rehabilitation

All patients were instructed to use crutches to limit weight-bearing for 2 weeks. Daily passive range-of-motion exercises were begun at postoperative Day 1. At 3 weeks, active range-of-motion and full weight-bearing activities were commenced. After 6 weeks, strengthening and light treadmill walking exercises were begun. Oral anti-inflammatory medication was taken daily for 4 weeks.

We collected data on continuous and categorical demographic and clinical variables, including age, sex, body mass index (BMI), duration of symptoms (from the onset of pain to operation) and smoking history during surgery.

**Fig. 1.**
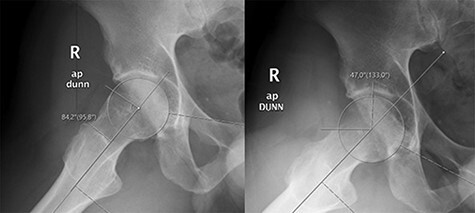
Preoperative and postoperative Dunn 45° views and measurement of alpha angle.

Radiographs were obtained and evaluated for all patients using the anteroposterior supine pelvis Dunn 45°X-rays. Osteoarthritis was graded using the Tönnis classification at preoperative and last follow-up pelvis X-rays [26]. LCEA was measured using the method described by Wiberg [25]. The alpha angle and femoral head-neck offset [27] were measured using preoperative and early postoperative Dunn 45° views (confirmed by measuring the degree of flexion by goniometer) (Fig. 1) [23, 24]. All measurements were made by consensus with the senior author (O.H.).

PROs, including the mHHS [28] and Visual Analog Scale for Pain (Pain VAS), were collected by contacting the patients by telephone (*n* = 10) or direct contact (*n* = 42) and from the medical records. Pain VAS and the mHHS were recorded on the day before the surgery and at the last follow-up assessment. Nwachukwu *et al*. [29] reported 5-year patient-acceptable symptomatic state (PASS) values after hip arthroscopy for FAI to be 83.6 for the mHHS. The minimal clinically important difference was not used because patients had been assessed at different time intervals.

## STATISTICAL ANALYSIS

The Shapiro–Wilk test was used initially to test for normality of distribution. Categorical variables were compared using the Fisher exact test. Paired *t*-test was used to assess differences between pre- and postoperative data. One-way analysis of variance was used to assess the effect of labrum treatment on the change in the mHHS or the last score. Preoperative to postoperative changes in VAS or Tönnis grade were assessed using the Wilcoxon test. The Spearman correlation analysis was then used to analyze the correlation between parameters.

The receiver operating characteristic curve (ROC) and area under the curve (AUC) were analyzed to further determine discriminatory threshold values for postoperative alpha angle or the change in the alpha angle (postoperative − preoperative) or head-neck offset distance to the frequency of reaching fifth-year PASS values for the mHHS or grade of osteoarthritis. An AUC between 0.70 and 0.80 was reported to be acceptable discrimination. To increase the reliability of Tönnis grading [30] and find a possible correlation with postoperative alpha angle and degree of postoperative osteoarthritis, we used simplified binary Tönnis classification and grouped the patients into two (Tönnis 0 + 1: Group1 and Tönnis Grade 2 + 3: Group 2) for the analysis.

The statistical significance was set at *P* < 0.05. Statistical analysis was performed with IBM SPSS Statistics for Windows, Version 24.0 (IBM Corp., Armonk, NY).

## RESULTS

Fifty-two unilateral hip arthroscopy patients were included. Patient demographics are given in Table I. The mean age of the patients was 33.9 years. The mean follow-up time was 6.7 years (5–8.6 years). Most patients received labral repair (74.6%) compared with labrum debridement.

**Table I.** Demographic data of the patients

*Patients*	*Mean ± SD(min–max)*
Age, years	33.9 ± 11.5 (16–61)
Sex	
Female	19 (36.5%)
Male	33 (63.5%)
Follow-up time, years	6.7 ± 0.9 (5–8.6)
Duration of symptoms, months	12 (3–48)
BMI	25.9 ± 3 (17.3–33.4)
Smoking	32 (61.5%)
Labrum treatment	
Debride	8 (15.4%)
Repair	
1 anchor	18 (34.6%)
2 anchors	20 (38.5%)
3 anchors	6 (11.5%)

SD, standard deviation.

The mean 45° Dunn view alpha angle decreased from 83.9 ± 4° to 49.7 ± 6.1° (*P* < 0.05) and anteroposterior LCEA decreased from 37.5 ± 7.9° to 36.3 ± 6.9° postoperatively. Anterior head-neck offset was increased from 2.3 ± 1.5 mm to 7.9 ± 2.1 mm postoperatively (*P*  < 0.001) (Table II).

**Table II.** Radiological parameters and functional scores

	*Preoperative*	*Postoperative*	*P-value*
Alpha angle, °	83.9 ± 4	49.7 ± 6.1	<0.001
LCEA, °	37.5 ± 7.9	36.3 ± 6.9	0.01
Head-neck offset (mm)	2.3 ± 1.5	7.9 ± 2.1	<0.001
Tönnis grade, *n* (%)			<0.001
0	32 (61.5)	23 (44.2)	
1	19 (36.5)	18 (34.6)	
2	1 (1.9)	9 (17.3)	
3	–	2 (3.8)	
Heterotopic ossification	–	8 (15.4%)	–
mHHS	60.1 ± 13.4	86.8 ± 14	<0.001
Pain VAS (IQR)	6 (2.5)	2 (2.5)	<0.001

The degree of osteoarthritis was increased at the final follow-up (preoperative Tönnis grade median ‘0’ to median ‘1’ postoperatively (*P* < 0.001). The degree of increase or the postoperative grade of osteoarthritis was not correlated with preoperative or postoperative alpha angle (*P*  > 0.05).

Postoperative mHHS only had a correlation with the grade hip osteoarthritis (*r* = −0.35; *P* = 0.01). Postoperative mHHS or change in mHHS was not correlated with any other parameters (age, BMI, smoking, sex, symptom duration, follow-up time, preoperative Tönnis grade, preoperative and postoperative alpha angles, LCE angles, head-neck offset distances, labrum treatment and heterotopic ossification (*P* > 0.05).

The mHHS improved from 60.1 ± 13.4 before surgery to 86.8 ± 14 after surgery (*P* < 0.001). The Pain VAS decreased from 6.21 (interquartile range [IQR] = 2.5) before surgery to 2 (IQR = 2.5) after surgery (*P* < 0.001).

Overall, 69% of the patients achieved the threshold value for PASS for mHHS at the final follow-up.

**Fig. 2.**
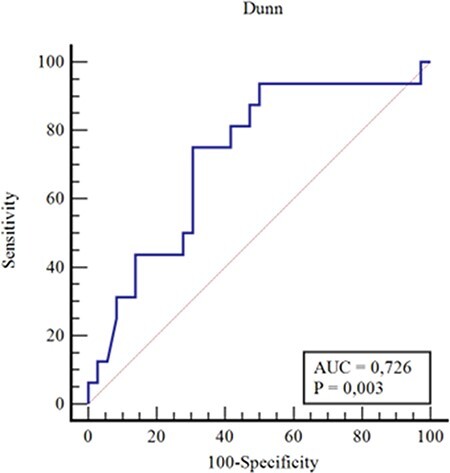
ROC curve for PASS alpha angle.

The ROC curve for postoperative alpha angle demonstrated acceptable discrimination between patients achieving the fifth-year PASS value and those who did not have an AUC of 0.72. Using the Youden index, a threshold of 48.3° was determined for the postoperative alpha angle. Patients having a postoperative alpha angle ≤48.3° achieved the fifth-year PASS value at a significantly higher rate than patients having a postoperative alpha angle >48.3° (*P* = 0.002, sensitivity 0.75 and specificity 0.69) (Fig. 2). There was no acceptable discrimination for head-neck offset value (*P* > 0.05) for the achievement of the fifth-year PASS value. Although the cutoff value of 33° for the change in alpha angle (the amount of decrease) was found to have a statistical significance (*P* = 0.02, sensitivity 0.58 and specificity 0.81), the AUC was below the acceptable value (0.67). Twenty-three of hip arthroscopy patients (44%) (14 men and 9 women) had a postoperative alpha angle of ≤48.3°, and 11 hip arthroscopy patients achieved a PASS value.

**Fig. 3.**
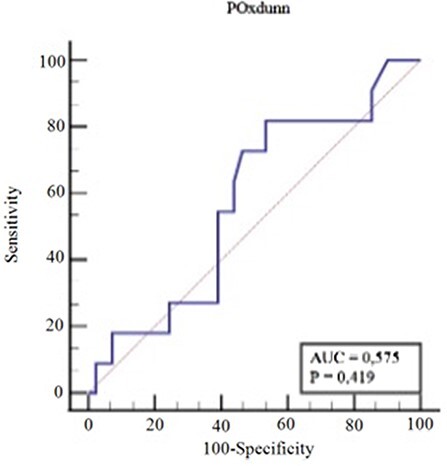
ROC curve for osteoarthritis (Tönnis Grade 2 + 3) alpha angle.

There was also no discriminative value of postoperative alpha angle to differentiate between osteoarthritic patients (Group 2: Tönnis Grade 2 + 3) and non-osteoarthritic patients (Group 1: Tönnis Grade 0 + 1) (*P* > 0.05) (Fig. 3).

## DISCUSSION

The main findings of this study were that 69% of the patients achieved the reported fifth-year PASS value for the mHHS, which is similar to the values reported in the literature (65.8–72.1%) [21, 22, 29]. Also, the postoperative alpha angle of ≤48.3° is associated with a higher frequency of reaching the PASS value, proving the hypothesis that categorization of numerical data in binary fashion led us to define a cutoff value for the postoperative alpha angle. In another word, while postoperative mHHS was only dependent on the last follow-up grade of hip osteoarthritis, when patients were grouped in binary fashion into two groups for the achievement of the fifth-year PASS score for the mHHS, ≤48.3° of postoperative alpha angle was found to be statistically significant to achieve the PASS score of 83.6 with a sensitivity of 0.75 and specificity of 0.69. There is a controversy about this issue in the literature. Briggs *et al*. [8] grouped patients into two having the postoperative alpha angle of <55° or ≥55°, and both groups had a median value of 85 points at the mHHS at a minimum follow-up of 5 years with no difference in any outcome measure. The postoperative alpha angle was at a wide range (30–100°). Similar to this, Kierkegaard *et al*. [11] also reported that a change in alpha angle was not correlated with 1-year PRO after hip arthroscopy. The alpha angle was reported to decrease from 51.7° to 46.8°. They cite this as a limitation that they did not have a large proportion of patients with very large alpha angles.

Lastly, Kaplan *et al*. [10] reported no association of postoperative alpha angles (preoperative 62° and postoperative 54°) at 2-year mHHS. Instead, they described a new FAI resection (FAIR) arc on 45° Dunn view radiograph similar to head-neck offset in the present study but placing a circle which is in contact with the anterior inferior iliac spine and the base of the lateral femoral neck. Maximal radial height (MRD) is then measured postoperatively. They demonstrated that patients in the cam MRD <3.15-mm group had significantly higher mHHS than those in the >3.15-mm group. However, we could not demonstrate that the effect of head-neck offset on the studied PROs. Thıs might be due to the technical difficulty of reproducibly measuring head-neck offset unlike the MRD described previously.

Contrary to those reports, two other studies reported the association between postoperative alpha angle and postoperative score or frequency of return to sports. Lansdown *et al*. [1] reported that preoperative and postoperative false-profile alpha angles were correlated with 2-year mHHS; the present study could not find a correlation, especially with preoperative alpha angles, probably due to longer-term follow-up but found a correlation of the postoperative alpha angle with categorical (yes or no for the fifth-year PASS achievement instead of numerical values) outcomes and also with probable additional non-linear and non-monotonic relation of alpha angle. Additionally, they did not specifically cite a cutoff value for the postoperative alpha angle. Only the study by Monahan *et al*. [15] reported that athletes with a postoperative alpha angle of ≤46° returned to sports at significantly higher rates than those with a postoperative alpha angle of >46°. The present study reported a bit higher cutoff value of 48.3° due to different expressions of outcomes such as the achievement of the fifth-year PASS value of the older patient group instead of return to sports at a younger athletic group and shorter follow-up (2 years).

A second controversial issue is whether correcting the alpha angle prevents the development of osteoarthritis. Forster-Horváth *et al*. [17] reported that pre- or postoperative Tönnis classification did not correlate with functional scores or radiological indices at a minimum of 55-month follow-up; they tried to describe this as fair-to-moderate reliability of Tönnis grading, especially at less-advanced cases [31]. We attempted to counteract this via binary grouping with non-osteoarthritic (Group 1: Tönnis Grade 0 + 1) and osteoarthritic (Group 2: Grade 2 + 3) patients, which has been shown to increase the intraobserver reliability from fair to excellent [30]. Similarly, Haefeli *et al*. [18] did not report any factor for failure (defined as conversion to total hip arthroplasty or progression of hip osteoarthritis ‘one or more Tönnis grades’ or poor clinical outcome) for treatment of arthroscopic FAI surgery at minimum of 5 years at 52 hips at a mean age of 35(16–63).

A study by Vahedi *et al*. [19] correlated the failure of femoroacetabular osteoplasty with a higher postoperative alpha angle (59.5° versus 56.6°). However, they defined the failure as conversion to total hip arthroplasty without defining the effect on functional scores or Tönnis grade, and both failure and non-failure groups had high alpha values.

The present study could not find a cutoff point for the postoperative alpha angle to discriminate between osteoarthritic (Tönnis Grade 2 + 3) and control groups (Tönnis Grade 0 + 1). Recently, Schmaranzer *et al*. [32] observed that a paradoxical decline in Delayed gadolinium-enhanced MRI of cartilage (dGEMRIC) index ‘increased cartilage degeneration’ 1 year after joint-preserving hip surgery but to a much lesser degree in symptomatic patients without surgical treatment for FAI despite clinical improvement in functional scores at 1 year postoperatively. They tried to explain this issue that the surgical intervention possibly altered the dGEMRIC properties of the cartilage by induction of the inflammation cascade, iatrogenic injury of the cartilage, surgical overcorrection and/or alteration of joint biomechanics or slow recovery potential of the bradytrophic articular cartilage covering several years.

Our study also did not find the effect of age, preoperative symptom duration, BMI, sex and the labrum treatment, of which controversial results have been reported for all in the literature [9, 17, 18, 22, 33, 34]. A lack of morbidly obese patients, > 2-year preoperative symptoms and a low number of labral debridement (8 versus 44 hip arthroscopy patients) patients may have affected the detection of statistical significance [22, 33].

There exist some limitations. First, the alpha angle as measured on the Dunn radiograph is an oversimplified two-dimensional view of cam impingement not using a three-dimensional evaluation of cam morphology using computed tomography (CT) [35]. Prior literature has established that the 45° Dunn view is most likely to provide the better visualization of the anterosuperior femoral neck, where most cam lesions are located compared with various radiograph projections and axial CT or magnetic resonance images [36–39]. Second, we used only the mHHS and Pain VAS as PROs, and other outcome scores like hip outcome score or international hip outcome tool-12 were not used. Third, we did not grade the severity of chondrolabral lesions. However, we have taken the study type of labrum treatment, number of anchors used and measured indirect signs of cartilage damage like Tönnis grading and preoperative alpha angle [38, 40, 41]. Fourth, this is a single-surgeon case series study with a small patient cohort without a control group and adjustment of confounding factors and with retrospective analysis of prospectively gathered data. Fifth, radiographic measurements were performed by a single observer although approved by the senior author, and interobserver or intraobserver reliability was not investigated. High interobserver errors could be made using a plain radiograph, and single reading may not be enough [42]. Sixth, there were a high number of patients excluded due to various reasons including revisions (*n* = 7) and failing to reach out (*n* = 13), which might have changed the results if they were also included. Last, certain factors including femoral version, psychiatric and back pain history were not controlled for and may have potentially influenced outcomes.

The present study failed to show an association between grade of osteoarthritis and postoperative alpha angle. Th’s failure might be because of possible low reliability of modified Tönnis grading and/or other factors that were overlooked in the present study prohibited the effect of alpha angle.

Other osteoarthritis grading systems may better enable cutoff values for binary classification of the degree of osteoarthritis by facilitating the detection of probable non-monotonic, non-linear relation with a postoperative alpha angle with ROC analysis like detected here for PASS values. Another clinically relevant question is whether there is also a lower limit for postoperative alpha angle for optimal functional scores. These two subjects are the topics of future studies.

## CONCLUSION

Overall, 69% of the patients with unilateral symptomatic FAI treated with hip arthroscopy achieved a fifth-year PASS value of the mHHS similar to that reported in the literature. The postoperative alpha angle of ≤48.3° is associated with a higher frequency of reaching the PASS value. Threshold of ≤48.3° had a sensitivity of 0.75 and a specificity of 0.69 to predict positivity. The postoperative alpha angle is a predictor of achievement of the fifth-year PASS value for the mHHS. However, future studies are needed to know whether there is a lower limit for postoperative alpha for optimal functional scores.

## Data Availability

The data that support the findings of this study are available on request from the corresponding author.
